# Shock Index and Characteristics of "Bounce-Back" Patients in the Emergency Department of King Abdullah Medical City (KAMC): A Retrospective Analysis

**DOI:** 10.7759/cureus.29692

**Published:** 2022-09-28

**Authors:** Abdulhameed O Alaama, Hawazen M Alsulaimani, Hadeel Alghamdi, Muruj M Alrehaili, Reham N Alsaud, Anwar M Almuqati, Nuha R Bukhari, Anas Alhassan, Noura M Bakhsh, Mohammad H Alwadei

**Affiliations:** 1 Emergency Medicine, King Abdullah Medical City, Makkah, SAU; 2 Medicine and Surgery, Umm Al-Qura University, Makkah, SAU; 3 Medical School, Umm Al-Qura University, Makkah, SAU; 4 Emergency Medicine, Umm Al-Qura University, Makkah, SAU; 5 Emergency Medicine, Security Forces Hospital, Makkah, SAU; 6 Emergency Medicine, King Fahad General Hospital, Makkah, SAU

**Keywords:** vital signs, shock index, saudi arabia, emergency, bounce back

## Abstract

Background

"Bounce back" patients is a term used to refer to patients returning to the emergency department within 72 hours after the first visit. This can be attributed to various factors related to diagnosis, management, or the health care system.

Objective

This study sought to evaluate the extent of bounce-back patients in the emergency department of King Abdullah Medical City (KAMC), Makkah, Saudi Arabia, and then explore the possible relationship between shock index (SI) and bounce-back patients.

Methods

This is a retrospective chart review of the electronic system among patients who have returned to the emergency department within 72 hours from the index visit. All records were reviewed from May 2019 to May 2021. Vital signs were collected to calculate the shock index (heart rate/systolic blood pressure). The data were analyzed by SPSS Statistics v.27.0 (IBM Corp., Armonk, NY).

Results

A total of (506) responses were analyzed. The median age was 56 years with an IQR of 40-67, and males represented 55.3%. Around three-quarters of the second complaints (76.9%) were related to the index visit. The durations between the visits were as follows: 51.8% within 24 hours, 30.2% within 25-48 hours, and 18% within 49-72 hours. The median and IQR for shock index were 0.67 and 0.59-0.80 respectively, while the median and IQR for reverse shock index were 1.49 and 1.25-1.71 respectively. Diabetes and the duration between the two visits were associated with the complaints (p-value=0.005, p-value=0.011) respectively.

Conclusion

The majority of bounce-back cases occurred within the first 24 hours in our sample. Hypertension, diabetes, and ischemic heart diseases were the most prevalent comorbidities among the bounce-back patients. The majority of bounce-back patients (76.9%) presented with complaints related to the index visit.

## Introduction

"Bounce back" is a term referring to patients returning to the emergency department within 14 days after the index visit. This definition includes a more extended period, which can give misleading results regarding bounce back [1].

In a study from California, the factors associated with bounce-back patients were chronic kidney disease, end-stage renal disease, congestive heart failure, and Medicaid insurance [2]. However, another study indicated that there is no relationship between bounce back and emergency crowding [3].

A retrospective study in California found that bounce-back patients were commonly from the African American community. While it can be considered a risk factor, the same ethnicity was associated with higher discharge rates which may indicate the association between higher rates of discharge and bounce back regardless of the demographic characteristics of the patients [4].

The shock index is a heart rate divided by systolic blood pressure and is predictive of the severity of the hypovolemic shock, which predicts mortality. For this reason, the shock index has been proven to be a good indicator for vasopressor administration. The shock index could predict cardiovascular collapse for sepsis patients [5].

A systemic review evaluating the benefits of shock index in some cases, including trauma, hemorrhage, myocardial infarction, pulmonary embolism, sepsis, and ectopic pregnancy, concluded that vital signs could give a false connotation about the patient's condition due to the compensatory phase. That study reported that a shock index of more than 1, gives an indication of high-risk patients, and increases the risk for mortality and morbidity, while a shock index of more than (0.7) may increase the probability of admission and mortality.

A retrospective study involving patients aged more than 65 years old studied the readmission rates within seven days according to blood pressure, heart rate, temperature, and oxygen saturation and found that patients discharged with one abnormal measure were not admitted within seven days [6]. Moreover, a cross-sectional study was done to investigate the need for activation of the trauma team and found that cases who did not meet the criteria for the trauma team and had a shock index less than “1” needed emergency critical care only [7].

It was found that the number of bounce-back patients has been constantly increasing at King Abdullah Medical City (KAMC), which raised our attention to investigate this issue. We aimed to study the characteristics of bounce-back patients in KAMC. We also evaluated their shock index. Our study results will give healthcare professionals insight into predicting mortality in bounce-back patients.

## Materials and methods

Study design

This is a retrospective study conducted among patients who returned to ER between 9 May 2019 to 9 May 2021 in King Abdullah Medical City (KAMC) in Makkah, Saudi Arabia. The study used a data collection sheet that was filled by two investigators from the electronic system. 

Study population 

The cohort of this study included bounce-back patients. The study population includes all the patients who returned to ER within 72 hours from the index visit. 

The inclusion criteria were adults of all genders who were discharged from ER at KAMC and returned within 72 hours, regardless of the complaint at the second visit. The exclusion criteria were children under 18 years old, deceased patients, and patients referred to the emergency from another facility. 

Data collection

Data were collected from patients’ electronic/files onto (paper/electronic) data collection sheets not showing any nominative information. Patients were identified by serial numbers and initials which are linked to the patients' name and medical record number (MRN) in a separate identification log sheet which was kept in a safe locked place. Two different persons performed data entry. After verification, data were transferred to a statistical database for analysis. Collected data included sociodemographic characteristics, comorbidities, vital signs, complaints during the two visits, and the dates of ER visits. The dates of visits were used to calculate the number of days to apply the inclusion criteria of bounce back within 72 hours of the index visit. 

Statistical analysis 

SPSS Statistics v.27.0 (IBM Corp., Armonk, NY) was used to analyze the data. Proportions were used to summarize categorical variables. The shock index was calculated by dividing the heart rate by the systolic blood pressure. The Shapiro-Wilk test was used to assess the normality of continuous variables distributions. All the continuous variables were not normally distributed, for which median and interquartile range (IQR) were used to summarize the data. Age was further categorized according to quartiles to allow comparison between similar sample sizes across age groups. The chi-square test was used to find significant associations between categorical variables. The significance level was set at (0.05).

Ethical considerations

Ethical approval was obtained from the research committee at King Abdullah international medical research center (KAIMRC) prior to data collection. All the collected data were kept with the primary author to maintain the privacy of the patients. After assigning serial numbers and linking them to the MRN, the data were transferred for data analysis. All the collected data were used for research purposes only.

## Results

The total analyzed responses were 506. The median and IQR for age were 56 and 40-67 years and males represented 55.3%. The patients' comorbidities included ischemic heart diseases, diabetes, hypertension, renal impairment, cancer, chronic pulmonary diseases, and other comorbidities were inquired. The details of demographic and medical characteristics are shown in Table 1.

**Table 1:** Demographic characteristics and comorbidities

Variables	Groups	N	%
Age	Less than 40	123	24.3%
	40-55	128	25.3%
	55-70	178	35.2%
	More than 70	77	15.2%
Gender	Male	280	55.3%
	female	226	44.7%
Comorbidities	Ischemic heart disease	130	25.7%
	Diabetes mellitus	216	42.7%
	Hypertension	263	52.0%
	Renal impairment	54	10.7%
	Cancer	101	20.0%
	Chronic pulmonary disease	45	8.9%
	Other	201	39.7%

The vital signs recorded during the first visit were collected. Heart rate, respiratory rate, oxygen saturation, body temperature, and blood pressure were analyzed and presented in Table 2. Using the heart rate and systolic blood pressure, the shock index (heart rate/systolic blood pressure) and reverse shock index (systolic blood pressure/heart rate) were calculated. The median and IQR rates for the shock index were 0.67, 0.59-0.80, while the median and IQR rates for the reverse shock index were 1.49, 1.25-1.71.

**Table 2:** Vital signs recorded at the first visit and the calculated shock indices

Variable	Minimum	Maximum	Median	Interquartile range (IQR)
Heart rate	43	185	85	76-98
Respiratory rate	11	34	18	18-19
O2 saturation	82	115	98	96-98
Body temperature	35	39.9	36.8	36.4-37.1
Blood pressure systolic	75	230	125	113-140
Blood pressure diastolic	46	153	72	65-80
Shock index	0.25	1.45	0.67	0.59-0.80
Reverse shock index	0.69	4	1.49	1.25-1.71

All the participants bounced back to the emergency department within three days of the index visit. The relationships between the complaints were assessed individually for each patient to determine the outcome variable groups as "related" and "not related". Around 76.9% of the second complaints were related to the index visit. The durations between the visits were as follows: 51.8% within 24 hours, 30.2% within 25-48 hours, and 18% within 49-72 hours.

Further analysis showed a significant association between diabetes and the duration of the two visits with the relation between complaints in the two visits (p-value=0.005, p-value=0.011) respectively. Other variables were tested for significance and did not show any significant associations (Table 3). Further analysis to find significant associations between the related complaints in the two visits with shock index and other variables did not reveal any significance.

**Table 3:** Associations with related complaints of the two visits

Variable		Are the two complaints related?				
		Yes		No		P-value
		Count	%	Count	%	
Age	Less than 40	22	17.9%	101	82.1%	0.373
	40-55	29	22.7%	99	77.3%	
	55-70	45	25.3%	133	74.7%	
	More than 70	21	27.3%	56	72.7%	
Gender	Male	61	21.8%	219	78.2%	0.427
	female	56	24.8%	170	75.2%	
Ischemic heart disease	Yes	33	25.4%	97	74.6%	0.478
	No	84	22.3%	292	77.7%	
Diabetes mellitus	Yes	63	29.2%	153	70.8%	0.005
	No	54	18.6%	236	81.4%	
Hypertension	Yes	66	25.1%	197	74.9%	0.274
	No	51	21.0%	192	79.0%	
Renal impairment	Yes	12	22.2%	42	77.8%	0.868
	No	105	23.2%	347	76.8%	
Cancer	Yes	24	23.8%	77	76.2%	0.865
	No	93	23.0%	312	77.0%	
Chronic pulmonary disease	Yes	7	15.6%	38	84.4%	0.207
	No	110	23.9%	351	76.1%	
Other comorbidities	Yes	54	26.9%	147	73.1%	0.105
	No	63	20.7%	242	79.3%	
Duration between visits	1-24 hours	53	20.2%	209	79.8%	0.011
	25-48 hours	32	20.9%	121	79.1%	
	49-72 hours	32	35.2%	59	64.8%	

## Discussion

This study evaluated the characteristics of bounce-back patients and the shock index used in the emergency department of King Abdullah Medical City (KAMC), Saudi Arabia. To our knowledge, this is the first study conducted evaluating both the bounce back and shock index. Our study results will give healthcare professionals insights into the shock index among bounce-back patients.

Most participants were middle-aged and male, which means that most bounce-back patients were among people in this age and gender group. Our findings are consistent with another study conducted at King Fahad Hospital, Saudi Arabia [8] and another study [9] that assessed demographics, and comorbidities in Saudi Arabia, which found that participants who were male and middle-aged were predominant.

Hypertension was the most prevalent comorbidity, followed by diabetes mellitus. Comorbidities have been established as predictors of bounce-back by other studies and the association of cardiovascular diseases with high bounce-back rates has been identified [8, 10-11]. Alshahrani et al. found that the most prevalent comorbidities were respiratory disease [8]. This might be due to their sample that included pediatric patients who are prone to frequent respiratory diseases. However, in line with our findings, they reported hypertension as the second most common comorbidity. Another study conducted on patients with COVID-19 showed that most patients who returned to the ER had pulmonary, cardiac, or infectious symptoms [12].

The majority of bounce-back patients (51.8%) presented within the first 24 hours after initial discharge, around a third were within 25-48 hours and the rest within 49-72 hours. This can be explained by the emergency nature of hypertension, diabetes, and ischemic heart diseases, which were the most prevalent comorbidities among returning patients. These conditions can exacerbate quickly and therefore need immediate care, which might have prompted patients to return as soon as possible. One previous cohort study found that most back-bounce visits within 24 hours were related to blood pressure, with over half (52%) being caused by persistent hypertension [13]. Our findings also align with another study that reported more return visits of patients to the ER for hypoglycemia, most of them being patients on oral diabetes medications [14]. Another study reported that there was a correlation between chest pain, which is one of the symptoms of ischemic heart disease, and return visits among cardiovascular disease patients, which might be the symptom of ischemic heart disease [15].

More than three-quarters of second complaints were related to the index visit. Diabetes (p=0.005) and the duration between two visits (p=0.011) were significantly associated with the similarity between the complaints during the initial and return visits. More returning patients with second symptoms related to the initial visits’ symptoms (29.2% vs 18.6%) had diabetes and returned within 49-72 hours. A study carried out by Betten et al. found that most diabetic patients who returned to the ER came within 48 hours and had hypoglycemia requiring readmission [14]. Among the complications of hypoglycemia and diabetes in general, there are cardiovascular disease and cardiac arrhythmia, which might lead to hypertension, ischemic heart diseases, and renal diseases. These conditions have been identified among our study participants. These conditions are known to be related and can affect one another [16-18], increasing the likelihood of return visits of patients with multiple morbidities.

Studying risk factors for recurrent ER visits of diabetic patients with hyperglycemia, Yan et al. [19] found that they constituted 18.7 of all return visits, and history of hyperglycemia, age < 2 years old were associated with more recurrent ER visits. Returning for hyperglycemia with the same history on the initial visits indicates the relationship between symptoms on return visits and previous symptoms, which is similar to our findings. In contrast, we didn’t find a significant association with age.

In general, diabetic patients with frequent episodes of hyperglycemia and sometimes hypoglycemia are expected to return to the ER frequently because they are, most of the time, the ones with poor control of glycemia levels [20].

Even if hypertension was the most prevalent, our calculated shock index median IQR was 0.67, ranging from 0.59-0.80, which includes the mortality predictive value of 0.7 [21]. On the other hand, IQR for blood pressure was not alarming. This result indicates that some patients were at risk of death despite their heart rates and blood pressure not being recorded as suspicious. However, patients had multiple comorbidities. This should be considered in addition to the diastolic pressure, which is not used by the shock index.

The shock index has been used by many studies to predict the prognosis and mortality among emergency and critically ill patients. In our study, the shock index among bounce-back patients was >1. While there were no previous studies addressing the association between shock index and the bounce back or readmission, a shock index of more than one was associated with higher mortality rates among patients admitted to the medical intensive care unit [22]. During the pandemic of COVID-19, the shock index was also used to assess the mortality among COVID-19 patients; this along with older age and oxygen saturation were found to be significant predictors of mortality [23]. The utility of shock index was also studied in triage for trauma patients, ectopic pregnancy, sepsis, myocardial infarction, and pulmonary sepsis. The strength of the shock index is represented in the feasibility of its calculation upon patient arrival. However, in the aforementioned conditions, a shock index of >0.7 was associated with higher admission and mortality rates [24].

There are some limitations for consideration. This study was retrospective and this may result in selection bias and missing data. In addition, this study was conducted in the ER only, making the results difficult to generalize to all patients. The absence of a control group presented the study results in a descriptive manner. Extensive, follow-up, and analytic studies are recommended for further exploration of bounce back in healthcare facilities.

## Conclusions

According to the findings of this study, abnormal shock index readings have limited predictive ability for bounce-back patients, while a reverse shock index could be a good predictor. 

This study showed more than half of bounce-back cases occurred within the first 24 hours and involved a larger number of male and middle-aged patients. Hypertension, diabetes, and ischemic heart diseases were the most common comorbidities among participants. More than three-quarters of return visit complaints were related to complaints presented during initial visits. Diabetes and duration were significantly associated with the relation between complaints in the initial and return visits.

Our findings highlight the need for measures to minimize bounce back by focusing on patients with the common morbidities identified. This study also opens opportunities for more research on the subject for all patients visiting healthcare facilities, especially overall bounce-back rates. We recommend studies on the bounce back to attending healthcare providers, as well as comparing bounce back rates among hospital departments. Studies evaluating bounce-back rates for all patients and associated factors would help establish strategies for improvement and compare them with other existing data.
